# Psychological measures of stress and biomarkers of inflammation, aging, and endothelial dysfunction in breast cancer survivors on aromatase inhibitors

**DOI:** 10.1038/s41598-023-28895-4

**Published:** 2023-01-30

**Authors:** Anne H. Blaes, Chandini Nair, Susan Everson-Rose, Patricia Jewett, Jack Wolf, Beshay Zordoky

**Affiliations:** 1 Department of Experimental and Clinical Pharmacology, University of Minnesota, Minneapolis, USA; 2 Department of Medicine, University of Minnesota, Minneapolis, USA; 3 Masonic Cancer Center, University of Minnesota, Minneapolis, USA; 4 Hematology/Oncology/Transplantation, University of Minnesota, Minneapolis, USA

**Keywords:** Cancer, Psychology, Oncology, Signs and symptoms

## Abstract

The use of aromatase inhibitors (AIs) is associated with higher rates of cardiovascular events and lower endothelial function in breast cancer survivors. Psychosocial stress is associated with higher levels of inflammatory and aging markers, and lower endothelial function in otherwise healthy subjects. These associations among breast cancer survivors on AIs are not well defined. A cross-sectional study of 30 breast cancer survivors on AIs was performed to assess the associations between self-reported scores of psychosocial measures of depression, anxiety, and stress assessed by validated questionnaires with markers of inflammation (CRP; IL-6; IL-18), aging (p16^INK4a^), and endothelial function (ICAM-1, EndoPAT ratio). Significant positive correlations were observed between psychosocial measures and inflammatory markers including CRP, IL-6, and ICAM-1. However, no psychosocial scores were related to endothelial function or gene expression of the aging biomarker p16INK4a. Overall, survivors had endothelial dysfunction with reduced EndoPAT ratios. Psychosocial stress is associated with greater inflammation in breast cancer survivors on AIs, corroborating previous studies in cancer-free populations. The lack of association between psychosocial stress and either endothelial function or aging biomarkers could be due to the already low endothelial function and accelerated aging in our cohort of breast cancer survivors on AIs, though our small sample size limits conclusions. Further work in a larger and more diverse cohort of patients is needed to further understand the relationships among inflammation, aging and endothelial function in breast cancer survivors.

## Introduction

Breast cancer (BC) is the world's leading cause of cancer mortality in women^1^. Approximately 1 in 8 women in the U.S. will be diagnosed with breast cancer in their lifetime. Although breast cancer is a leading cause of cancer mortality worldwide, the 5- and 10-year survival rates are 90% and 84%, respectively^2^. This is due in large part to early detection and improved treatment methods, including extended treatment with aromatase inhibitors (AIs), the primary adjuvant endocrine therapy for breast cancer survivors with estrogen positive tumors^3^. Although AIs reduce the risk of breast cancer recurrence, extended use of AIs is associated with increased risk of cardiovascular disease events. Aromatase catalyzes the final step in the biosynthesis of estrogen and reduced endogenous estrogen levels leading to a loss of estrogen-mediated protection from endothelial dysfunction and cardiovascular disease in postmenopausal women^4,5^. The vascular protective effects of estrogen are mediated via enhanced nitric oxide (NO) signaling^6^ and anti-inflammatory effects^7^, among others.Recent evidence suggests adverse changes in vascular function begin to occur almost as soon as AI treatment begins^8^.

Receiving a breast cancer diagnosis, subsequent treatments and symptom management can be a stressful and life-changing experience^9^. Indeed, the psychosocial impacts of breast cancer are well recognized, with higher rates of depression and anxiety observed among breast cancer survivors compared to the general female population, even 5 years post-diagnosis^10,11^. Depression and other indicators of both acute and chronic stress are associated with increased levels of circulating inflammatory and aging markers as well as changes in endothelial function in otherwise healthy subjects^12–14^. However, these associations among breast cancers survivors on extended AI treatments are not well understood.

To address this gap in the literature, the current study evaluated cross-sectional associations among three indicators of psychosocial stress (depression, anxiety, and perceived stress), markers of inflammation and aging, and endothelial function among a cohort of 31 breast cancer survivors on AI recruited to participate in a pilot randomized control trial (RCT) of mindfulness based stress reduction. Analyses reported herein were limited to the baseline data obtained as part of that RCT, prior to the intervention.

## Methods

### Subjects

A cohort of 30 breast cancer survivors on AIs were recruited to participate in a pilot randomized control trial (RCT) of mindfulness-based stress reduction. Individuals with a diagnosis of hormone receptor positive HER2 negative breast cancer who were prescribed an aromatase inhibitor at the University of Minnesota Masonic Cancer Center were eligible. All cases had completed breast cancer treatment and were actively taking an AI. Subjects with a history of tobacco use, myocardial infarction, congestive heart failure, or cardiac catheterization requiring intervention were excluded. Medical record abstraction for cases confirmed diagnosis, stage at diagnosis, use of chemotherapy, use of radiation, and personal medical history including medications (including type of prescribed aromatase inhibitor). Participants completed surveys, laboratory and vascular testing. The protocol was approved by the University of Minnesota Institutional Review Board and the Masonic Cancer Center Review Committee. All methods were performed in accordance with the relevant guidelines and regulations. All patients provided written informed consent according to the Declaration of Helsinki.

### Study recruitment

Potential cases were identified by their treating physician and were then invited to participate in the study between June and August 2020. For those who expressed interest, a screening phone call was placed to discuss the study and identify participants. Failure to answer the phone call or return the phone call was identified as passive refusal to participate in the study. Consented subjects were invited to undergo vascular assessment and biomarker assessment at baseline and completion of the study. Those consented subjects who completed the protocol received one $25 gift card. In total, 43 individuals were screened for the study, 33 met eligibility and enrolled, 30 had evaluable data. Most participants were diagnosed with stage 1 (n = 12; 40.0%) or stage 2 (n = 15; 50.0%) breast cancer, with just 3 participants (10.0%) diagnosed with stage 3. Among the study participants, 13 were on letrozole, 11 on anastrazole, and 6 on exemestane. Median time on AI was 33 months (range 12–76).

### Vascular assessment

Vascular assessment was performed using the EndoPAT 2000 as previously described^5,15,16^. EndoPAT measures the dilatory response through pulsatile volume changes at the fingertips, known as Peripheral Artery Tone (PAT). EndoPAT technology is a non-invasive FDA-approved testing device for the diagnosis of endothelial dysfunction. The EndoPAT examination required the patient to be supine, at rest, in a quiet, air-conditioned room. Subjects were fasting at least 8 h before the study. Subjects were asked not to exercise, use caffeine, high-fat foods, or vitamin C for at least 4–6 h before the test. Low EndoPAT ratios (< 1.6) are indicative of worsening endothelial function and increased CV risk^5^.

All study assessments were conducted in a specifically designed research space for clinical vascular assessments. Blood collection and vascular functional measurements were conducted in the morning by a trained technician. All testing was completed by the same technician to reduce inter-technician variability. The EndoPAT assessments were performed in triplicate, and the mean (average of 3 values for each participant) indices were used in statistical analyses.

### Biomarker assessment

Biomarkers were obtained using a fasting blood draw to evaluate the following inflammatory markers: C-reactive protein (CRP), interleukin-6 (IL-6), and interleukin-8 (IL-18). Prior research has shown that CRP, IL-6 and IL-18 are three key inflammatory biomarkers associated with inflammation in breast cancer survivors^17,18^. ICAM-1 is a cell surface glycoprotein expressed at low basal level in endothelial, epithelial, and immune cells, but is upregulated in response to inflammation^19^. ICAM-1 has been reliably used as a biomarker for endothelial dysfunction^20^. Cellular senescence was assessed by measuring the expression of CDKN2A, the gene encoding p16^INK4a^ in CD3 + peripheral blood mononuclear cells (PBMCs) as a surrogate biomarker for human aging^21,22^. The Translational Therapy Laboratory (TTL) purified CD3 + PBMCs from bulk PBMCs isolates from whole blood samples. Total RNA was isolated from patient CD3 + PBMCs and CDKN2A/p16^INK4a^ and 18S housekeeping gene expressions were measured by real-time quantitative polymerase chain reaction by TTL staff. Blood samples were collected first, prior to vascular functional assessments. All blood samples were appropriately processed and aliquoted for measurement of respective biomarkers.

### Psychosocial measures

The following questionnaires were completed by participants at baseline: The 4-item CDC Healthy Days measure evaluating self-reported health and quality of life^23^, PHQ-9 Depression Scale^24^, Perceived Stress Scale^25^, and Generalized Anxiety Disorder^26^. The 4-item CDC Healthy Days measure assesses quality of life by asking about self-reported health (1 item; response options ranging from poor to excellent) and the number of days in the past month that physical health was poor, mental health was poor, and daily activities were impacted by either poor physical or mental health (3 items)^23^. The self-administered Patient Health Questionnaire (PHQ)-9 is a validated clinical and research tool to measure depression symptoms and severity^24^. Item responses range from “0” (not at all) to “3” (nearly every day) for a combined scoring of 0–27(scores of 5–9 are classified as mild depression; 10–14 as moderate depression; 15–19 as moderately severe depression; ≥ 20 as severe depression. The Perceived Stress Scale (PSS) measures the degree to which situations in one’s life are appraised as stressful^25^. The 10 item PSS is suggested for examining the role of nonspecific appraised stress and as an outcome measure of experienced levels of stress, with scores ranging from “0” (never) to “4” (very often). Scores ranging from 0–13, 14–26, 27–40 would be considered low, moderate, or high perceived stress, respectively. The GAD-7 is a clinical measure for assessing generalized anxiety disorder (GAD). The questionnaire consists of seven items that are summed to generate a global score^26^; cut points of 5, 10, and 15 can be interpreted as representing mild, moderate, and severe levels of anxiety.

### Statistical methods

Analyses reported were limited to the baseline data obtained as part of the RCT, prior to the intervention, and thus represent a cross-sectional evaluation. Descriptive statistics were calculated across all subjects as mean (SD) for continuous variables and count (percentage) for categorical variables. Continuous variables that were highly skewed were log10 transformed before any statistical modeling was performed. One negative EndoPAT measurement was coded as NA and omitted from all analyses. Outcome measurements were assumed to be missing completely at random. All regression models used a complete-case analysis and subjects with a missing value for either the independent or dependent variable were removed. We estimated the mean increase in each inflammatory or senescence marker associated with a one point increase in each psychosocial score (GAD, PSS, and PHQ) through three independent simple regression models for each inflammatory or senescence marker. We reported point estimates of the associated average increase along with 95% confidence intervals. All significance testing was performed at the 5% level. No adjustments for multiple comparisons were made. All analyses were conducted using R v3.6.1 (R Core Team, 2019).

The datasets used and/or analysed during the current study are available from the corresponding author on reasonable request.

## Results

### Patient characteristics

The demographic characteristics of study participants (n = 30) are listed in Table 1. Of the participants, 46.7% (n = 14) had received both chemotherapy and radiation treatments, 33.3% (n = 10) had received only radiation treatment, 13.3% (n = 4) had received only chemotherapy treatment, and 6.7% (n = 2) had received neither chemotherapy nor radiation treatment. The average age of the subjects was 59.0 (SD = 10.5) years. Of the participants, 96.8% (n = 30) were white, and 93.5% (n = 29) had completed either college or graduate/professional training. All participants were postmenopausal women with estrogen positive breast cancer. They had a baseline cancer stage 1 (n = 12), stage 2 (n = 15), or stage 3 (n = 3).

**Table 1:** Demographic summary of the study population.

Covariate	Overall mean values (N = 30)
Age—mean (SD)	59.5 (10.3)
Race	
White	29 (96.7%)
Asian	1 (3.3%)
Ethnicity	
Not Hispanic or Latina	24 (80.0%)
Missing ethnicity	6 (20.0%)
Education	
Graduate or professional training	13 (43.4%)
College graduate	15 (50.0%)
Some college/technical school	2 (6.7%)
Bassline cancer stage	
Stage 1	12 (40.0%)
Stage 2	15 (50.0%)
Stage 3	3 (10.0%)
Treatment	
Chemotherapy, radiation	14 (46.7%)
Chemotherapy, no radiation	4 (13.3%)
No chemotherapy, radiation	10 (33.3%)
No chemotherapy, no radiation	2 (6.7%)

Average EndoPAT ratio, psychosocial stress measure scores, inflammatory biomarker concentrations, and senescence biomarker concentrations for the study population as a whole are displayed in Table 2. The EndoPAT ratio (mean of 0.60) was low in all participants where low EndoPAT ratios (< 1.6) were previously associated with worsening endothelial function and increased CV risk^5^. Mean scores from the PHQ, GAD, and PSS assessments indicate that subjects had low levels of depression, anxiety, and stress. In our sample, we calculated Cronbach’s alpha values of 0.766 for the PHQ-9, 0.823 for the GAD-7, and 0.841 for the PSS. Values for all measures could not be obtained from all study participants and the number of subjects missing any one of these values is also shown in Table 2.

**Table 2:** Covariate measures for the study population.

Covariate	Overall mean (SD) or median [IQR] values
EndoPAT ratio (N = 26)	0.6 (0.31)
PHQ score (N = 29)	3.5 (3.1)
GAD score (N = 30)	2.9 (2.7)
PSS score (N = 28)	11.5 (6.9)
CDC healthy days measure	
General health (N = 30)	3.7 (0.74)
Days with physical health issues (N = 30)	0.0 [0.0, 2.0]
Days with mental health issues (N = 30)	2.0 [0.0, 4.8]
Days kept from usual activities (N = 30)	0.0 [0.0, 0.75]
Change in Ct (N = 28)	25.0 (1.2)
C-reactive protein mg/L (N = 30)	3.0 (2.3)
IL-6 pg/mL (N = 28)	1.5 [0.92, 2.35]
IL-18 pg/mL (N = 30)	168 (63.8)
Soluble I-CAM1 ng/mL (N = 30)	232.1 [204.7, 275.7]

Values are mean (SD) or median [IQR] for numeric covariates where the median is used for highly skewed variables.

**Figure 1:**
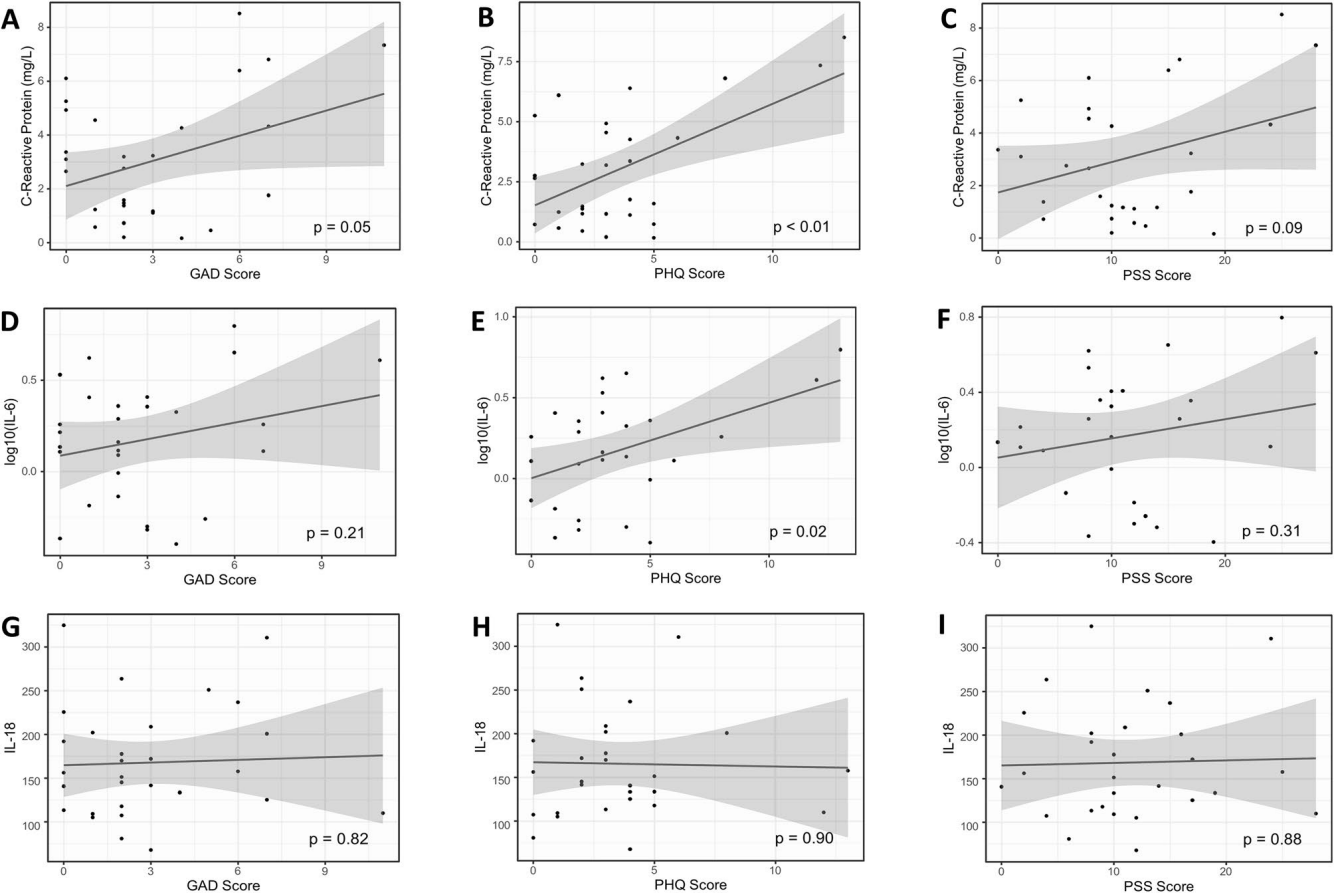
Associations between psychosocial stress scores and inflammatory marker concentrations.

Associations between psychosocial stress scores and inflammatory marker concentrations are shown in Fig. 1. No subjects met the criteria for acute inflammation/infection. The highest CRP was < 10 mg/L. Higher GAD (β^^^ = 0.31 (0.00, 0.63); p = 0.05) and PHQ scores (β^^^ = 0.42 (0.17, 0.67); p < 0.01) were significantly and positively correlated with higher CRP levels as shown in Fig. 1A and B. A pattern of positive association between higher PSS scores and CRP levels was also noted, but the association was not statistically significant (β^^^ = 0.12 (− 0.02, 0.25); p = 0.09). Higher PHQ scores were significantly (p < 0.05) associated with increased IL-6 concentrations (β^^^ = 0.05 (0.01, 0.09); p = 0.02) (Fig. 1E). While a trend of positive association was observed between higher GAD scores (Fig. 1D; (β^^^ = 0.03 (− 0.02, 0.08); p = 0.21)) and PSS scores (Fig. 1F; (β^^^ = 0.01 (− 0.01, 0.03); p = 0.31)) and higher levels of IL-6, neither of these associations were statistically significant. There was no significant association between psychosocial stress scores and IL-18 levels (Supplemental Table 1).

**Figure 2:**
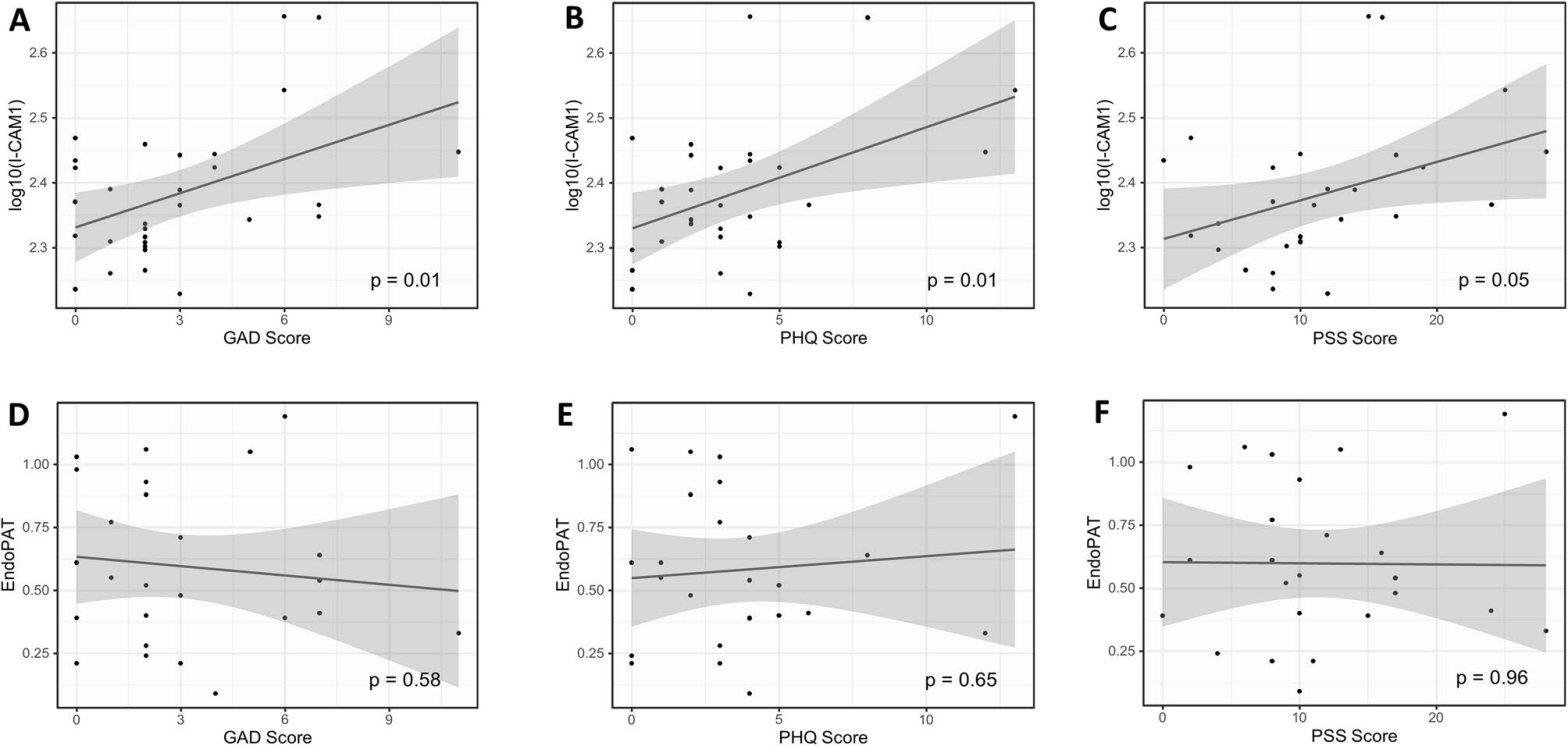
Correlation of endothelial dysfunction markers with psychosocial stress measures.

Associations between psychosocial stress scores and endothelial dysfunction are shown in Fig. 2. Surface expression of ICAM-1, an intercellular adhesion molecule, is increased in states of inflammation and endothelial dysfunction and it can serve as a risk marker for future coronary events^27^. There were significant positive associations between ICAM-1 with higher GAD (β^^^ = 0.02 (0.00, 0.03); p = 0.01), PHQ (β^^^ = 0.02 (0.00, 0.03); p = 0.01), and PSS (β^^^ = 0.01 (0.00, 0.01); p = 0.05) scores as seen in Fig. 2A–C. However, actual measurements of endothelial dysfunction using EndoPAT did not translate into significant associations between EndoPAT ratios and higher psychosocial stress scores (Fig. 2D–F).

**Figure 3:**
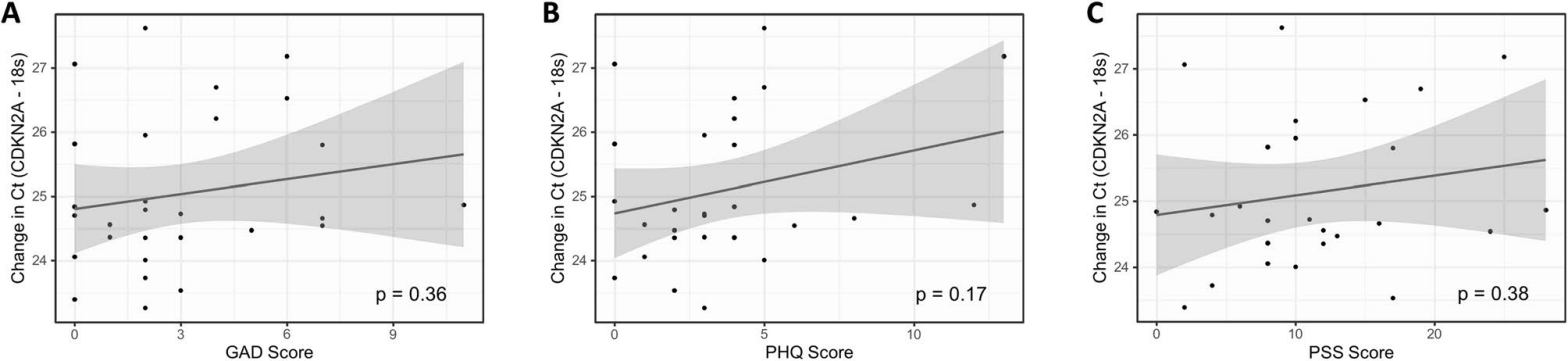
Correlation of senescence markers with psychosocial stress measures.

Activation of the CDKN2A gene by tissue damage or cellular stress induces the expression of p16, a common biomarker of cellular senescence^21^. The Ct (cycle threshold), as shown in Fig. 3, is the number of amplification cycles required to cross a fixed background level of fluorescence upon measuring gene expression by real-time PCR. The Ct values are inversely proportional to the gene expression (i.e. higher gene expression leads to lower Ct values). The Ct values reported in the figure were the measure of change in the count of CDKN2A normalized to those of 18S as a housekeeping gene. The associations between psychosocial stress scores and change in Ct were not statistically significant, indicating psychosocial stress scores were not associated with CDKN2A gene expression.

## Discussion

Our findings in breast cancer patients on AIs demonstrate a significant positive correlation between psychosocial stress scores and several inflammatory markers, corroborating previous studies in cancer-free populations. Nevertheless, in contrast to studies in otherwise healthy subjects, there was no significant association between scores of perceived stress, depression, or anxiety and either endothelial function or aging biomarkers. In this cohort of breast cancer survivors on aromatase inhibitors, endothelial dysfunction was present, consistent with previous reports^28^.

Survivorship of breast cancer patients has remarkably improved over the past three decades, thanks to advancements in diagnostics, care models, and therapeutics including aromatase inhibitors (AIs). AIs are the standard treatment for postmenopausal patients with hormone receptor-positive breast cancer as both adjuvant and metastatic treatments^29^. It is estimated that 5 years of an AI compared with no endocrine therapy would reduce breast cancer recurrence by about two-thirds during treatment and by about one-third during years 5–9^30^. Despite their superior efficacy over tamoxifen for hormone-sensitive breast cancer^31^, aromatase inhibitors may increase the risk of cardiovascular diseases, including hypertension, dyslipidemia, and ischemic heart disease^32,33^.

While the prognosis for breast cancer is generally positive, breast cancer survivors are affected by tremendous physical, functional, financial, emotional, and social challenges^9^, which creates significant psychosocial stress. The prevalence of depression is highest during the first year after a breast cancer diagnosis, and according to a systematic review, the frequency of depressive symptoms ranges from 9.4 to 66.1%^10^. Other studies have found a 42% rate of psychiatric disorders in recently treated breast cancer survivors and 35.7% of these women had depression, anxiety, or both^11^. Additional systematic reviews have suggested a higher prevalence of depression and anxiety among breast cancer survivors when compared to the general female population, even more than 5 years after diagnosis^10^. Nonetheless, we note that our patient sample had relatively low levels of depressive symptoms, anxiety and perceived stress at the time of their study enrollment. Our participants were, on average on AIs for 33 months suggesting they may have learned to cope well with their diagnosis and concomitant treatment. Additionally, psychosocial stress may be low in these women given that most of them had been diagnosed with early stage breast cancer, with excellent prognoses. Regardless, we did observe positive associations between psychosocial measures and most of our inflammatory biomarkers, suggesting a clear relationship between inflammation and psychosocial functioning.

Side effects of AIs such as vasomotor complaints, vaginal dryness, and diminished libido may also add to the higher stress levels in breast cancer survivors^9,34^. Breast cancer survivors taking AIs are more likely to report sexual problems than those taking tamoxifen^35^ or not having any endocrine therapy^36^.

Inflammatory pathways contribute to the pathophysiology of both cancer and cardiovascular disease^37^. Several studies have previously examined inflammatory markers as predictors of disease recurrence and death in cancer populations. Cancer survivors with persistent inflammation may have an elevated risk of recurrence as a result of the effects of inflammatory processes^38^. Three key inflammatory biomarkers associated with inflammation in breast cancer survivors are CRP, IL-6, and IL-18. CRP is a nonspecific protein produced by the liver in response to inflammation, infection, and tissue damage. CRP concentrations in women diagnosed with breast cancer are positively associated with death due to any cause, death due to breast cancer, and additional breast cancer events^38^. The association between IL-6 elevation and increased risk of cancer recurrence is less clear and may only apply to certain breast cancer subtypes such as HER2- tumors^39^. Studies have shown that breast cancer survivors with elevated IL-18 serum levels have shorter relapse-free survival durations than those with lower IL-18 levels^40^.

Higher levels of inflammatory cytokines were reported in the blood of breast cancer survivors by several groups^38,41–43^. Inflammatory cytokines increase to a varying degree following radiation^44^ and chemotherapy^41,45^. Significantly higher IL-6 among depressed cancer patients compared to healthy subjects, and higher levels of inflammatory markers were associated with fatigue and sleep disturbance^41,44,46^. Similarly, higher levels of IL-6 are associated with frailty in older breast cancer survivors^47^. Several studies have also reported the correlation between inflammatory mediators and measures of psychosocial stress in breast cancer survivors. The inflammatory mediators IL-6 and TNF-alpha were positively correlated with social anxiety and depressive symptoms, respectively in two studies of breast cancer survivors^48,49^. In breast cancer survivors one year post-radiotherapy, both depressive symptoms and higher inflammatory markers were independent risk factors of fatigue; however, the correlation between depressive symptoms and inflammatory markers was not statistically significant^49,50^. CRP was positively associated with depressive symptoms only among women who reported high levels of cancer-related stress in a longitudinal study of women with breast cancer^51^. Inflammation was positively correlated with depressive symptoms only in women who also reported high level of anxiety, perceived stress, negative affect, or sleep disturbance^52^. Counterintuitively, IL-6 was found to negatively correlate with anxiety symptoms in one study^49^. In agreement with the majority of these studies, in our cohort of breast cancer survivors on AIs, depressive symptoms were significantly and positively correlated with CRP and IL-6 with similar, albeit non-significant patterns of associations noted for anxiety and perceived stress. In contrast, IL-18 was not correlated to psychosocial measures in our study.

Several lines of evidence suggest that the associations between psychosocial stress and inflammation exist in cancer-free subjects as well^12^. Chronic psychosocial stress may induce heightened inflammatory activity through the hypothalamic–pituitary–adrenal axis (HPA) and the autonomic nervous system^53^. Psychosocial stress has been associated with higher circulating cytokines levels including TNF-alpha and IL-6^53^. In women, high levels of job stress and burnout were associated with high levels of TNF-alpha^13^ and CRP^14^. A strong inverse correlation was found between socioeconomic status and levels of circulating CRP, TNF-alpha and IL-6^54,55^. Loneliness was also associated with higher IL-6^56^. In addition to the observational studies that show a strong association between psychosocial stress and inflammation, experimental studies demonstrate a bidirectional cause-effect relationship between stress and inflammation. Intriguingly, acute laboratory stress paradigms show that stress can cause an inflammatory response^57,58^, and inflammation can also provoke depressive symptoms^59,60^.

Aromatase inhibitors and ionizing radiation have been linked with reduced endothelial function in cancer survivors^5,61^. Endothelial dysfunction, which is an independent risk factor for cardiovascular events in the Framingham risk score, precedes and potentially mediates the development of several cardiovascular diseases^15,62^. Reactive hyperemia measured non-invasively by EndoPat is a consistent surrogate for endothelial function in clinical studies^15^. Using the EndoPat technique, we have previously demonstrated reduced endothelial function in postmenopausal breast cancer survivors on an AI in comparison to healthy postmenopausal women^5^. In the current study, the average EndoPat value in BC survivors on AIs was 0.6 similar to the value reported in our previous study^5^. The association between psychosocial stress and endothelial dysfunction has not been reported in breast cancer survivors. In the current small cohort of BC survivors on an AI, there was no significant correlation between parameters of psychosocial stress and EndoPat values.

Psychosocial stress is associated with impaired endothelial function in several populations, including young adults with major depressive disorder^63^. Mental stress induces endothelial dysfunction in healthy subjects^64^. The lack of association between psychosocial stress and low EndoPat values in the current study may be due to the already low values in a cohort of breast cancer survivors on AIs and/or the small sample size of our study. ICAM-1 is a biomarker for endothelial dysfunction that belongs to a family of adhesion molecules, which are mediators of cellular adhesion between endothelial cells and leukocytes^20^. ICAM-1 is an independent risk factor for atherosclerosis and developing coronary heart disease^65^. Higher chronic stress was associated with lower flow-mediated dilation and higher ICAM-1^66^ and higher ICAM-1 was significantly higher in low socioeconomic status subjects^67^. In the current study, we observed a significant correlation between parameters of psychosocial stress and ICAM-1 in breast cancer survivors on AIs. The positive correlation between stress and ICAM-1 may be predictive of a later-onset decline in endothelial function if the stress continues.

Cancer survivors demonstrate clinical signs of premature aging and frailty^68^. In addition to the clinical evidence of premature aging, cancer survivors demonstrate high levels of aging biomarkers, such as *p16*^*INK4a*^ a marker of cellular senescence that is encoded by the *CDKN2a* gene^69^. In breast cancer patients, chemotherapy significantly increased the expression of *p16*^*INK4a*^, comparable with 10–17 years increase of chronological age^70,71^. Anthracycline-based chemotherapy was associated with the largest increase in *p16*^*INK4a*^ expression, comparable with 23–26 years of chronological age^71^. Intriguingly, aging-related functional limitations were reached 5–10 years earlier in breast cancer survivors than cancer-free women^72^. In the general population, chronic psychosocial stress was associated with increased expression of *p16*^*INK4a*^^73^. Nevertheless, the association between psychosocial stress and biomarkers of aging has not been specifically reported in breast cancer patients. In our cohort of breast cancer survivors on AIs, there was no significant correlation between psychosocial stress and *p16*^*INK4a*^.

The current study has a number of limitations that warrant discussion. First, the study lacks a comparison group of same-aged women without breast cancer. Therefore, we cannot infer whether the observed associations or the lack thereof is specific to breast cancer survivors on AIs or generalizable to an age-matched cancer-free population. Second, the small sample size may have hindered our ability to detect statistically significant correlations between multiples variables. Finally, our sample was predominantly white, well-educated women; thus, generalizability to other demographic groups could be limited.

In conclusion, our findings in breast cancer patients on AIs demonstrate a significant positive correlation between psychosocial stress scores and several inflammatory markers, corroborating previous studies in cancer-free populations. Nevertheless, in contrast to studies in otherwise healthy subjects, there was no significant association between psychosocial stress cores and either endothelial function or aging biomarkers. This observed lack of association may be due to the already low endothelial function and accelerated aging in our cohort of breast cancer survivors on AIs, or due to the small number of patients included in this study. Further work in a larger cohort of patients is needed to confirm or refute this lack of association.

## Supplementary Information

Supplementary Table 1.

## Data Availability

The datasets used and/or analysed during the current study available from the corresponding author on reasonable request.
