# Supplementary material for: Psychological measures of stress and biomarkers of inflammation, aging, and endothelial dysfunction in breast cancer survivors on aromatase inhibitors
Source: Sci Rep. 2023 Jan 30;13:1677. doi: 10.1038/s41598-023-28895-4 (PMC9886974; doi:10.1038/s41598-023-28895-4)
Supplement: Supplementary file 1 — Supplementary Table 1. [file 41598_2023_28895_MOESM1_ESM.docx]

**Supplemental Tables:**

| ***Outcome*** | ***Psychosocial Score*** | ***Effect Estimate*** | ***95% CI*** | ***P-Value*** |
| --- | --- | --- | --- | --- |
| *C-Reactive Protein* | GAD Score | 0.31 | (0.00, 0.63) | 0.05 |
|  | PSS Score | 0.12 | (-0.02, 0.25) | 0.09 |
|  | PHQ Score | 0.46 | (0.17, 0.75) | < 0.01 |
| *log10(IL-6)* | GAD Score | 0.03 | (-0.02, 0.08) | 0.21 |
|  | PSS Score | 0.01 | (-0.01, 0.03) | 0.31 |
|  | PHQ Score | 0.05 | (0.01, 0.10) | 0.02 |
| *IL-18* | GAD Score | 1.01 | (-8.15, 10.18) | 0.82 |
|  | PSS Score | 0.29 | (-3.55, 4.14) | 0.88 |
|  | PHQ Score | -1.43 | (-10.61, 7.75) | 0.75 |
| *log10(I-CAM1)* | GAD Score | 0.02 | (0.00, 0.03) | 0.01 |
|  | PSS Score | 0.01 | (0.00, 0.01) | 0.05 |
|  | PHQ Score | 0.02 | (0.00, 0.03) | 0.02 |
| *Change in Ct (CDKN2A - 18s)* | GAD Score | 0.08 | (-0.09, 0.25) | 0.36 |
|  | PSS Score | 0.03 | (-0.04, 0.10) | 0.38 |
|  | PHQ Score | 0.11 | (-0.06, 0.27) | 0.19 |
| *EndoPAT* | GAD Score | -0.01 | (-0.06, 0.03) | 0.58 |
|  | PSS Score | -0.00 | (-0.02, 0.02) | 0.96 |
|  | PHQ Score | 0.01 | (-0.04, 0.05) | 0.72 |

Table S1: Estimated effects of a one point increase of a given psychosocial score on a given marker of senescence or inflammation. Estimates were calculated through independent simple linear regression models.
